# Machine Learning–Based Prediction of Substance Use in Adolescents in Three Independent Worldwide Cohorts: Algorithm Development and Validation Study

**DOI:** 10.2196/62805

**Published:** 2025-02-24

**Authors:** Soeun Kim, Hyejun Kim, Seokjun Kim, Hojae Lee, Ahmed Hammoodi, Yujin Choi, Hyeon Jin Kim, Lee Smith, Min Seo Kim, Guillaume Fond, Laurent Boyer, Sung Wook Baik, Hayeon Lee, Jaeyu Park, Rosie Kwon, Selin Woo, Dong Keon Yon

**Affiliations:** 1 Center for Digital Health, Medical Science Research Institute, Kyung Hee University Medical Center Seoul Republic of Korea; 2 Department of Precision Medicine, Kyung Hee University College of Medicine Seoul Republic of Korea; 3 Department of Applied Information Engineering, Yonsei University Seoul Republic of Korea; 4 Department of Medicine, Kyung Hee University College of Medicine Seoul Republic of Korea; 5 Department of Business Administration, Kyung Hee University School of Management Seoul Republic of Korea; 6 Department of Korean Medicine, Kyung Hee University College of Korean Medicine Seoul Republic of Korea; 7 Centre for Health, Performance and Wellbeing, Anglia Ruskin University Cambridge United Kingdom; 8 Cardiovascular Disease Initiative, Broad Institute of MIT and Harvard Cambridge, MA United States; 9 Research Centre on Health Services and Quality of Life, Assistance Publique-Hopitaux de Marseille, Aix Marseille University Marseille France; 10 Department of Software, Sejong University College of Electronics and Information Engineering Seoul Republic of Korea; 11 Department of Electronics and Information Convergence Engineering, Kyung Hee University Seoul Republic of Korea; 12 Department of Pediatrics Kyung Hee University Medical Center Kyung Hee University College of Medicine Seoul Republic of Korea

**Keywords:** adolescents, machine learning, substance, prediction, XGBoost, random forest, ML, substance use, adolescents, adolescent, South Korea, United States, Norway, web-based survey, survey, risk behavior, smoking, alcohol, intervention, interventions

## Abstract

**Background:**

To address gaps in global understanding of cultural and social variations, this study used a high-performance machine learning (ML) model to predict adolescent substance use across three national datasets.

**Objective:**

This study aims to develop a generalizable predictive model for adolescent substance use using multinational datasets and ML.

**Methods:**

The study used the Korea Youth Risk Behavior Web-Based Survey (KYRBS) from South Korea (n=1,098,641) to train ML models. For external validation, we used the Youth Risk Behavior Survey (YRBS) from the United States (n=2,511,916) and Norwegian nationwide Ungdata surveys (Ungdata) from Norway (n=700,660). After developing various ML models, we evaluated the final model’s performance using multiple metrics. We also assessed feature importance using traditional methods and further analyzed variable contributions through SHapley Additive exPlanation values.

**Results:**

The study used nationwide adolescent datasets for ML model development and validation, analyzing data from 1,098,641 KYRBS adolescents, 2,511,916 YRBS participants, and 700,660 from Ungdata. The XGBoost model was the top performer on the KYRBS, achieving an area under receiver operating characteristic curve (AUROC) score of 80.61% (95% CI 79.63-81.59) and precision of 30.42 (95% CI 28.65-32.16) with detailed analysis on sensitivity of 31.30 (95% CI 29.47-33.20), specificity of 99.16 (95% CI 99.12-99.20), accuracy of 98.36 (95% CI 98.31-98.42), balanced accuracy of 65.23 (95% CI 64.31-66.17), *F*_1_-score of 30.85 (95% CI 29.25-32.51), and area under precision-recall curve of 32.14 (95% CI 30.34-33.95). The model achieved an AUROC score of 79.30% and a precision of 68.37% on the YRBS dataset, while in external validation using the Ungdata dataset, it recorded an AUROC score of 76.39% and a precision of 12.74%. Feature importance and SHapley Additive exPlanation value analyses identified smoking status, BMI, suicidal ideation, alcohol consumption, and feelings of sadness and despair as key contributors to the risk of substance use, with smoking status emerging as the most influential factor.

**Conclusions:**

Based on multinational datasets from South Korea, the United States, and Norway, this study shows the potential of ML models, particularly the XGBoost model, in predicting adolescent substance use. These findings provide a solid basis for future research exploring additional influencing factors or developing targeted intervention strategies.

## Introduction

Substance use among adolescents remains a global concern, often leading to both immediate and long-term health challenges, such as mental health disorders and addiction [1]. When initiated at an early age, these behaviors can escalate to more serious health conditions, including chronic substance dependence and comorbid mental health issues [2]. As globalization and cultural integration continue to expand, substance use patterns vary significantly across regions, making it paramount to understand these patterns across a diverse cultural landscape [3]. Conventional statistical methods have long been used to examine the predictors and outcomes of adolescent substance use [4]. However, these approaches often fall short in capturing complex, nonlinear relationships between variables. Therefore, with recent advancements, machine learning (ML) has introduced powerful tools capable of identifying complex patterns and relationships, offering a deeper understanding of adolescent substance use [5].

Existing studies provide insights into the epidemiology and sociocultural factors associated with adolescent substance use in various contexts, but few have used ML techniques with multinational datasets [4,6]. Such approaches have the potential to yield more precise and globally relevant insights [5,7]. By identifying key predictors of adolescent substance use that remain consistent across diverse cultural contexts, this study aims to develop a prediction model adaptable to global public health initiatives. To validate its generalizability, the model was tested using datasets from two additional countries, highlighting its adaptability to diverse sociocultural environments [8].

This study developed an ML-based prediction model for adolescent substance use, using comprehensive datasets from South Korea, and extended our validation process by using datasets from the United States and Norway [9]. This validation process and refinement process ensured the model’s accuracy and applicability across diverse cultural and national contexts. By integrating these global datasets, we developed a predictive model that reflects our collaborative international research. Our novel approach equips stakeholders with a sophisticated tool informed by global data. This helps address and preempt adolescent substance use effectively across different national contexts.

## Methods

### Study Design and Participants

Adolescents enrolled in middle and high school who completed their respective surveys were included. In the context of educational systems, adolescents are more appropriately categorized by grade level rather than age. To ensure consistency across the three countries, participants were limited to students from middle school (7th grade) to high school (12th grade). This study was primarily designed to develop an ML model for substance use prediction among adolescents using three distinct nationwide datasets: Korea Youth Risk Behavior Web-Based Survey (KYRBS) from South Korea [10], Youth Risk Behavior Survey (YRBS) from the United States [6,11], and Norwegian nationwide Ungdata surveys (Ungdata) from Norway [12]. KYRBS was initially used to train the ML model, followed by the external validation process using the YRBS and Ungdata.

**Figure 1:**
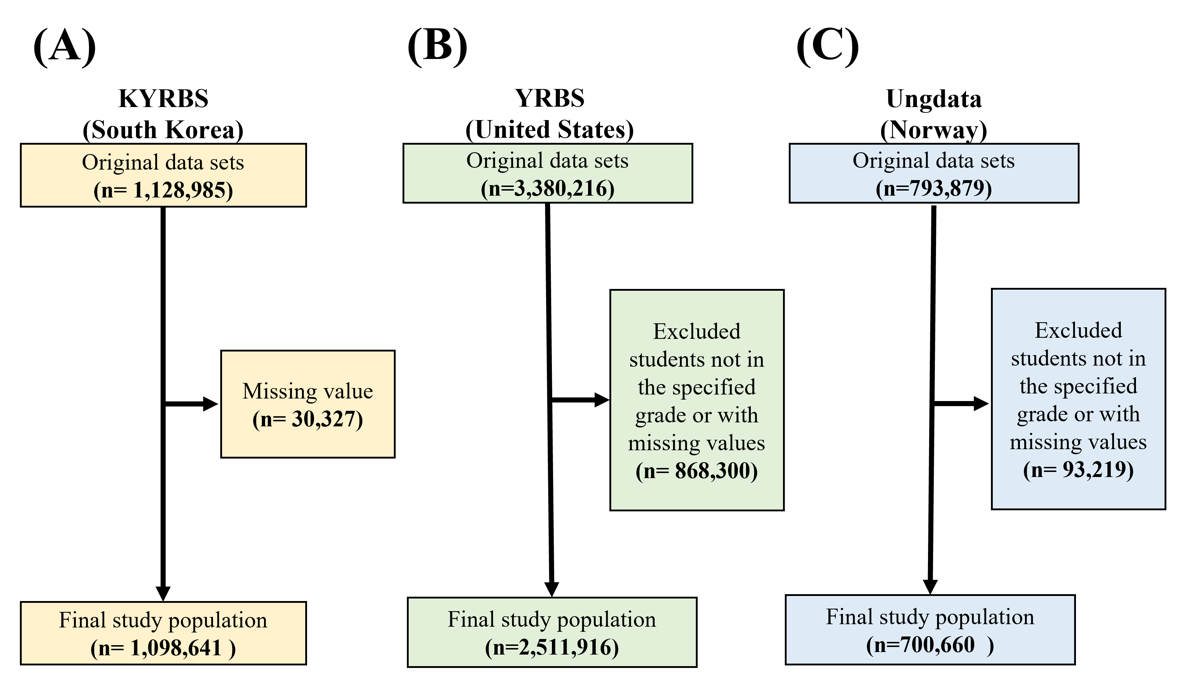
Study population. KYRBS: Korea Youth Risk Behavior Web-Based Survey; Ungdata: Norwegian nationwide Ungdata surveys; YRBS: Youth Risk Behavior Survey.

The discovery dataset, KYRBS, was conducted annually by the Korean Disease Control and Prevention Agency (KDCA) from 2008 to 2022, to assess health behaviors among Korean middle and high school students. It began with 1,128,985 participants and ended up with 1,098,641 after exclusions from the missing values in BMI [13], primarily representing a demographic largely comprised of East Asians (Figure 1A). The KYRBS dataset can be accessed through the official website of the KDCA.

The YRBS, which was conducted by the US Centers for Disease Control and Prevention (CDC), along with state and local education and health agencies, began with 3,380,216 participants, was narrowed down to 2,511,916 after excluding students not enrolled in middle or high school and those with missing data (Figure 1B**)**. YRBS encompasses a diverse racial spectrum of American adolescents. YRBS data are downloaded and available through the US CDC’s official website.

Similarly, from the initial count of 793,879 in the Ungdata, a cross-sectional survey conducted by the social research institute, Norwegian Social Research Institute (NOVA), at Oslo Metropolitan University consists of a questionnaire for school pupils throughout, only 700,660 participants were selected after data processing (Figure 1C). Ungdata is offered to all local and county councils in Norway, who administer the questionnaire in collaboration with NOVA and regional centers for substance use rehabilitation. The dataset can be accessed via the official Ungdata website. During the data processing phase, individual missing values in the KYRBS and YRBS datasets were imputed using a random forest regression–based imputation method [14]. For the extravalidation cohorts, YRBS and Ungdata, variables that were entirely absent were imputed using the median values derived from the discovery dataset, KYRBS.

In order to evaluate the generalizability of our model across diverse cultural groups, we used a phased validation approach. The validation process used the YRBS dataset, which includes participants from diverse ethnic backgrounds, as well as the Ung dataset from Norway, representing a markedly different cultural context. By using validation datasets encompassing a wide range of cultural groups, we conducted a rigorous assessment of the model’s robustness and versatility across diverse populations [15].

Our primary outcome, substance use, was derived from the question “Have you ever consumed illicit substances at least once in your lifetime.” We distinguished smoking and alcohol from other substances in our analysis, recognizing their unique consumption patterns, sociocultural implications, and health effects [16]. Substances other than smoking and alcohol are distinguished primarily due to concerns regarding their potential for misuse and health risks [17]. This decision was made to ensure that our model captures nuances specific to each substance, thereby enhancing the specificity and relevance of our predictions. Integral covariates under consideration spanned across factors including grade, sex, region, BMI, academic achievement, household income, smoking status, alcoholic consumption, stress status, sadness and despair, suicidal thinking, and suicide attempts [13].

### Model Development and Validation

Using the KYRBS, we developed a predictive model to extrapolate the behavioral patterns of Korean adolescents regarding substance use. Due to rigorous substance regulations and law enforcement measures in South Korea, accessibility and consumption of substances are notably limited [18]. Consequently, the number of instances representing substance use within the KYRBS was sparse (n=12,803, 1.17%).

Given the intricate nature of the data, we used a comprehensive approach to model development, leveraging 10-fold cross-validation to divide the KYRBS dataset into training and testing subsets. Various tree-based and statistical models, including LightGBM, CatBoost, AdaBoost, random forest, and XGBoost, were evaluated in comparison with logistic regression, a widely used baseline model, to determine the most effective algorithm for predicting substance use [19,20].

After model development using the KYRBS, external validation was conducted with datasets from diverse cultural contexts (YRBS and Ungdata) to evaluate the generalizability of the predictive model. This step ensured the applicability of the modeling approach across heterogeneous adolescent populations [21].

To further strengthen the validity of our results, hyperparameter tuning was performed for each algorithm using GridSearchCV, focusing on maximizing performance metrics such as the area under the receiver operating characteristic curve (AUROC) and precision. The hyperparameter values for the selected model are detailed in Table S1 in Multimedia Appendix 1. The sensitivity and specificity of the model were determined based on a classification threshold of 0.5. Various metrics, including AUROC score, accuracy, sensitivity, specificity, balanced accuracy, precision, *F*_1_-score (ie, the harmonic mean of the precision and recall), and the area under precision-recall curve (AUPRC) were used to evaluate the model’s performance across datasets [13].

### Performance Assessment

The tools and techniques we used for assessment were consistent. Our evaluation metrics comprised AUROC, accuracy, sensitivity, specificity, balanced accuracy, precision, *F*_1_-score, and AUPRC. These metrics were collectively considered to comprehensively evaluate and compare model performance. To provide a visual representation of the model efficacy, we used visualization techniques, notably the ROC curve [19,20,22-24].

### SHapley Additive ExPlanation Value Analysis

To interpret and better understand the model’s predictions, we calculated the SHapley Additive explanation (SHAP) values based on the summary model [25]. SHAP is a widely used method for providing local explanations of ML model predictions. Proposed by Lundberg and Lee, SHAP offers a unified framework for explaining the output of any ML model [25]. This technique visualizes the contribution of each feature to the model’s prediction for a specific instance, illustrating how each feature shifts the model’s output from the base value.

### Software and Libraries

All computations, model training, validation, and evaluation processes were executed using Python (version 3.12.4; Python Software Foundation). Key libraries from our toolbox included Scikit-learn (version 1.5.2; Scikit-learn development team), NumPy (version 1.26.4; Python Software Foundation), and Pandas (version 2.2.0; Python Software Foundation) for ML tasks and data wrangling. Visualization was facilitated using Matplotlib (version 3.8.4; Python Software Foundation) and Seaborn (version 0.13.2; Python Software Foundation).

### Ethical Considerations

The study protocol was approved by the institutional review board of the KDCA (2014-06EXP-02-P-A), US CDC (#1969.0), and NOVA (18778329) [12], and all participants provided written informed consent. This research followed the guidelines outlined in the TRIPOD (Transparent Reporting of a multivariable prediction model for Individual Prognosis or Diagnosis) statement [26].

## Results

### Demographic Characteristics

This research used a detailed exploration using nationwide adolescent datasets from South Korea, aiming to design and validate an ML model to predict substance use tendencies among adolescents. The primary demographic consisted of middle (7th grade) to high school (12th grade) students (Figure 1).

We collected data from the KYBS, the YRBS, and the Ungdata, and subsequently standardized the covariates for the ML predictive modeling process. Within the primary cohort from the KYRBS to develop the prediction model, the sex distribution was as follows: male (n=566,437, 51.56%) and female (n=532,204, 48.44%). For the initial external validation cohorts of an external validation process, the YRBS features a sex distribution of: male (n=1,233,846, 49.12%) and female (n=1,278,070, 50.88%). In the second validation step, the Ungdata has the following sex distribution: male (n=345,428, 49.30%) and female (n=355,232, 50.70%; Table 1).

**Table 1:** Demographic characteristics of KYRBS^a^ from South Korea (2005-2022), YRBS^b^ from the United States (1998-2022), and Ungdata from Norway (2014-2021).

		South Korea (KYRBS; n=1,098,641), n (%)						United States (YRBS; n=2,511,916), n (%)						Norway (Ungdata; n=700,660), n (%)											
**Region**																									
	Urban				509,058 (46.34)						N/A^c^						N/A								
	Rural				589,583 (53.66)						N/A						N/A								
**Grade**																									
	7th grade		190,112 (17.30)						448,865 (17.87)						144,229 (20.58)										
	8th grade	190,166 (17.31)						402,753 (16.03)						141,806 (20.24)											
	9th grade	189,842 (17.28)						476,360 (18.96)						143,938 (20.54)											
	10th grade	182,908 (16.65)						448,865 (17.87)						127,053 (18.13)											
	11th grade	181,428 (16.51)						402,753 (16.03)						89,637 (12.79)											
	12th grade	164,185 (14.94)						332,320 (13.23)						53,997 (7.71)											
**Sex**																									
	Male	566,437 (51.56)						1,233,846 (49.12)						345,428 (49.30)											
	Female	532,204 (48.44)						1,278,070 (50.88)						355,232 (50.70)											
**BMI^d^**																									
	Unknown			2940 (0.27)						448,630 (17.86)						N/A									
	Underweight			88,787 (8.08)						62,540 (2.49)						N/A									
	Normal			830,683 (75.61)						1,428,266 (56.86)						N/A									
	Overweight			89,971 (8.19)						308,141 (12.27)						N/A									
	Obese			86,260 (7.85)						264,339 (10.52)						N/A									
**Academic achievement**																									
	Low (0-19 percentile)			114,832 (10.45)						N/A						N/A									
	Lower-middle (20-39 percentile)			255,845 (23.29)						N/A						N/A									
	Middle (40-59 percentile)			314,304 (28.61)						N/A						N/A									
	Upper-middle (60-79 percentile)			278,359 (25.34)						N/A						N/A									
	High (80-100 percentile)			135,301 (12.32)						N/A						N/A									
**Household income**																									
	Low (0-19 percentile)			43,920 (4.00)						N/A						7968 (1.14)									
	Lower-middle (20-39 percentile)			159,283 (14.50)						N/A						27,885 (3.98)									
	Middle (40-59 percentile)			516,595 (47.02)						N/A						121,858 (17.39)									
	Upper-middle (60-79 percentile)			290,304 (26.42)						N/A						236,136 (33.70)									
	High (80-100 percentile)			88,539 (8.06)						N/A						306,813 (43.79)									
**Smoking status**																									
	Nonsmoker			869,907 (79.18)						1,179,077 (46.94)						562,118 (80.23)									
	Smoker			228,734 (20.82)						1,332,839 (53.06)						138,542 (19.77)									
**Alcoholic consumption**																									
	Nondrinker				581,345 (52.91)						1,335,962 (53.18)						306,210 (43.70)								
	More than one time				517,296 (47.09)						1,175,954 (46.82)						394,450 (56.30)								
**Stress status^e^**																									
	Low			30,456 (2.77)						N/A						N/A									
	Mild			159,720 (14.54)						N/A						N/A									
	Moderate			455,026 (41.42)						N/A						N/A									
	High			324,847 (29.57)						N/A						N/A									
	Severe			128,592 (11.70)						N/A						N/A									
**Sadness and despair in the past year**																									
	Unknown						N/A						1,079,817 (42.99)						N/A						
	No						749,479 (68.22)						1,005,251 (40.02)						522,702 (74.60)						
	Yes						349,162 (31.78)						426,848 (16.99)						177,958 (25.40)						
**Suicidal thinking in the past year**																									
	No					914,543 (83.24)						1,506,410 (59.97)						N/A							
	Yes					184,098 (16.76)						1,005,506 (40.03)						N/A							
**Suicide attempts in the past year**																									
	No		1,057,142 (96.22)						1,899,009 (75.60)						N/A										
	Yes	41,499 (3.78)						612,907 (24.40)						N/A											
**Substance use**																									
	No		1,085,838 (98.83)						1,310,191 (52.16)						685,866 (97.89)										
	Yes	12,803 (1.17)						1,201,725 (47.84)						14,794 (2.11)											

^a^KYRBS: Korea Youth Risk Behavior Web-Based Survey.

^b^YRBS: Youth Risk Behavior Survey.

^c^N/A: not applicable.

^d^BMI was divided into four groups according to the 2017 Korean National Growth Charts: underweight (0-4 percentile), normal (5-84 percentile), overweight (85-94 percentile), and obese (95-100 percentile).

^e^Stress was defined by the receipt of mental health counseling owing to stress.

We compared the distributions of key variables across the three cohorts (KYRBS, YRBS, and Ungdata), as presented in Table 1. Notable similarities and differences were identified. For instance, the proportion of smokers differed significantly between KYRBS (228,734, 20.82%) and YRBS (n=1,332,839, 53.06%), while the Ungdata cohort exhibited a much lower smoking prevalence (n=138,542, 19.77%). Similarly, alcohol consumption rates were higher in the Ungdata cohort (n=394,450, 56.30%) compared to KYRBS (n=517,296, 47.09%) and YRBS (n=1,175,954, 46.82%). These differences likely reflect cultural and policy variations influencing substance accessibility and behavioral norms in each country. Furthermore, visual comparisons of key baseline characteristics, such as smoking and alcohol consumption, are provided in Figure S1 in Multimedia Appendix 1 to offer additional insights.

### ML Model Results

**Figure 2:**
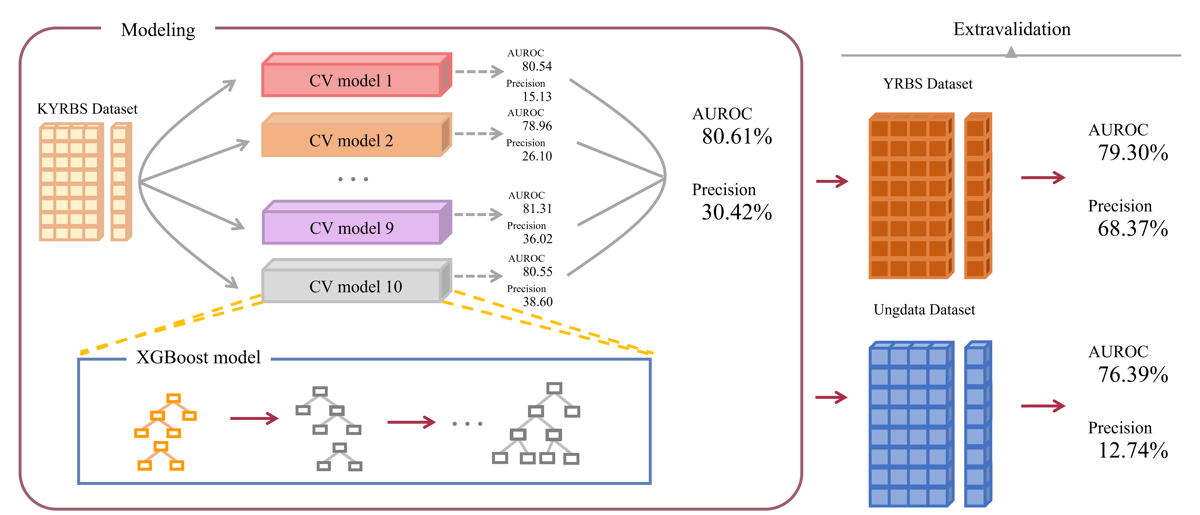
Model architecture. The original KYRBS was partitioned into the original data set for model development, with performance assessed using the AUROC score. Selected high-performing models were further validated. The external validations were generated using YRBS and Ungdata. AUROC: area under the receiver operating characteristic curve; KYRBS: Korea Youth Risk Behavior Web-Based Survey; YRBS: Youth Risk Behavior Survey.

**Figure 3:**
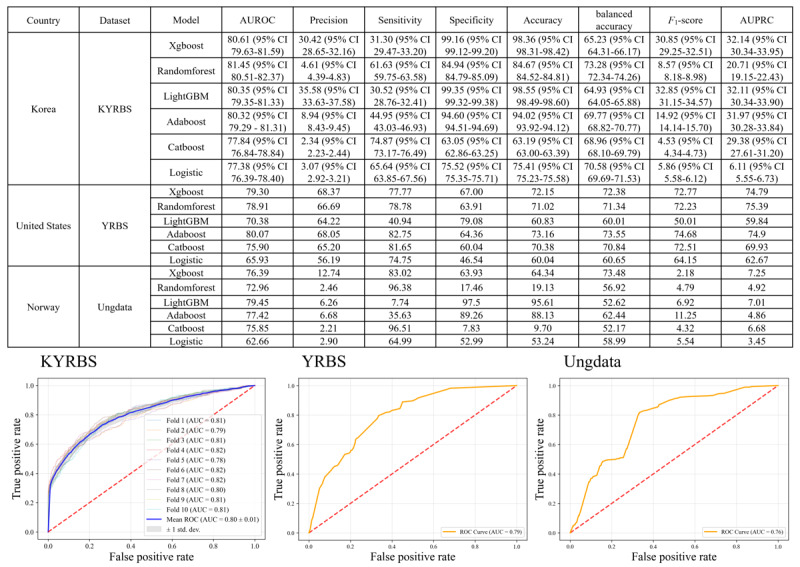
The assessment of five different machine learning algorithms using AUROC score and ROC curve for initial model construction with the KYRBS, and external validation with the YRBS and Ungdata. AUROC: area under the receiver operating characteristic curve; KYRBS: Korea Youth Risk Behavior Web-Based Survey; LightGBM: light gradient boosting model; ROC: receiver operating characteristic; XGBoost: extreme Gradient Boosting model; YRBS: Youth Risk Behavior Survey.

Extensive model evaluations, considering both AUROC and precision, revealed that the XGBoost model was the optimal model for predicting substance use among adolescents (Figures 2 and 3). The primary model, sourced from the KYRBS and assessed disclosed that the XGBoost model notched an AUROC score of 80.61% (95% CI 79.63-81.59) and precision of 30.42 (95% CI 28.65-32.16) with detailed analysis on a sensitivity of 31.30 (95% CI 29.47-33.20), specificity of 99.16 (95% CI 99.12-99.20), accuracy of 98.36 (95% CI 98.31-98.42), balanced accuracy of 65.23 (95% CI 64.31-66.17), *F*_1_-score of 30.85 (95% CI 29.25-32.51), and AUPRC of 32.14 (95% CI 30.34-33.95). Other models exhibited the following AUROC scores: random forest at 81.45 (95% CI 80.51-82.37), LightGBM at 80.35 (95% CI 79.35-81.33), AdaBoost at 80.32 (95% CI 79.29-81.31), CatBoost at 77.84 (95% CI 76.84-78.84), and Logistic 77.38 (95% CI 76.39-78.40). Precision scores were as follows: LightGBM at 35.58 (95% CI 33.63-37.58), AdaBoost at 8.94 (95% CI 8.43-9.45), random forest at 4.61 (95% CI 4.39-4.83), Logistic at 3.07 (95% CI 2.92-3.21), and CatBoost at 2.34 (95% CI 2.23-2.44).

For the initial external validation, the independent YRBS dataset was used. The XGBoost displayed an AUROC score of 79.30%, followed by a precision of 68.37%, sensitivity of 77.77%, specificity of 67%, accuracy of 72.15%, and balanced accuracy of 72.38%, *F*_1_-score of 72.77%, and AUPRC of 74.79%. In the subsequent external validation using the Ung dataset, the XGBoost model yielded an AUROC score of 76.39%, coupled with a precision of 12.74%, sensitivity of 83.02%, specificity of 63.93%, accuracy of 64.34%, balanced accuracy of 73.48%, *F*_1_-score of 2.18, and AUPRC of 7.25 (Figure 3).

Across all evaluations, both internal and external, the XGBoost model consistently exhibited a predominant performance, particularly in terms of the AUROC score and precision, cementing its adoption for the study objective.

### Feature Importance

Table 2 illustrates the importance of various features as determined by the XGBoost model in predicting substance use among adolescents. Specifically, smoking status was identified as the most significant predictor, accounting for 16.62% importance. This was closely followed by BMI with 13.45%, and suicidal thinking at 12.58%. Other notable features include suicide attempts (9.88%), grade (9.83%), stress status (8.99%), academic achievement (7.51%), household income (7.45%), sadness and despair (6.84%), alcoholic consumption (3.78%), sex (2.76%), and region (0.32%).

**Table 2:** Feature importance of the XGBoost model.

Feature	Importance (%)
Smoking status	16.62
BMI	13.45
Suicidal thinking	12.58
Suicide attempts	9.88
Grade	9.83
Stress status	8.99
Academic achievement	7.51
Household income	7.45
Sadness and despair	6.84
Alcoholic consumption	3.78
Sex	2.76
Region	0.32

### SHAP Values

**Figure 4:**
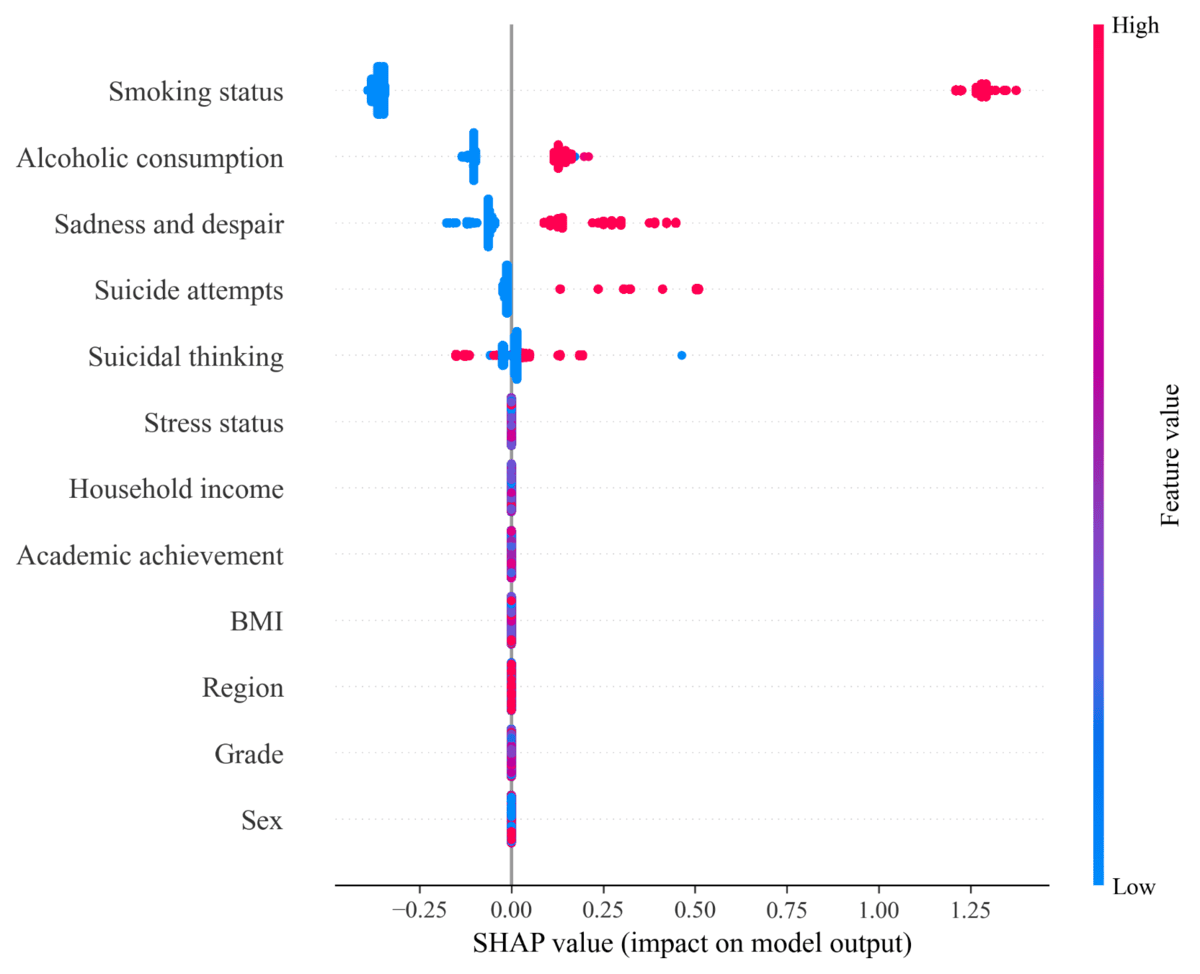
SHAP value of the XGBoost model. SHAP: SHapley Additive exPlanation.

Figure 4 presents the SHAP analysis results for the substance use prediction model, illustrating the contribution of each variable to predicting the likelihood of substance use [25]. The analysis identified smoking status as the most influential variable, with higher smoking levels strongly associated with an increased likelihood of substance use. Similarly, alcoholic consumption and sadness and despair were identified as key factors; alcohol consumption consistently increased the likelihood of substance use, while emotional states such as sadness exhibited both positive and negative effects depending on their levels.

Suicidal thinking and suicide attempts also showed distinct and significant impacts, with higher values substantially increasing the likelihood of substance use. In contrast, variables such as BMI, grade, and sex had relatively minimal contributions, indicating that the model is more sensitive to psychological and behavioral factors than to demographic characteristics. These findings offer critical insights into the primary predictors of substance use and their complex interactions.

### Code Availability

Based on the results of the ML model, we established a web-based application for policy implementation or health system management to support their decision-making process for cases involving substance use in adolescents [27]. An example of a web interface and the results are shown in Figure S2 in Multimedia Appendix 1. Custom code for the website is available on the web [28].

## Discussion

### Key Findings

This study stands out as one of the first comprehensive ML-based approaches to predict adolescent substance use on an international scale. One of our critical insights revolves around the influence of cultural diversity on substance use, drawing datasets from South Korea, the United States, and Norway [21]. Moreover, the outcome revealed that the XGBoost model proves to be commendable. It displayed predictive capabilities with an AUROC score of 80.61% (95% CI 79.63-81.59) and a precision of 30.42% (95% CI 28.65-32.16) in the discovery dataset. Our model consistently exhibited robust performance across external validation sets, achieving AUROC scores of 79.30% and 76.39% and precision of 68.37% and 12.74 in each respective dataset. Upon closer inspection, we discerned pivotal features influencing adolescent substance use predictions. Smoking status emerged as the predominant predictor for substance use, followed by BMI and suicidal thinking. Furthermore, the SHAP value analysis confirmed smoking status as a critical variable, with alcoholic consumption and sadness and despair identified as additional influential factors. To apply our findings to real-world scenarios, we devised a cutting-edge web-based platform. We believe that this tool will serve as an insightful methodology for the public to navigate potential substance-related challenges.

### Plausible Mechanism

The influence of smoking status and alcohol consumption on adolescent substance use is noteworthy and warrants consideration. Neurobiological evidence indicates that nicotine, especially when introduced during formative years, can alter the brain reward pathways, making other substances more appealing [29]. Similarly, alcohol can modify neurotransmitter levels during these formative years, making the brain more susceptible to effects from other substances [30,31]. Smoking and drinking during adolescence often signal risk-taking behaviors [32], leading to further experimentation with other substances.

Both smoking and alcoholic consumption align closely with societal expectations and peer pressures. Societal dynamics and peer interactions, which may normalize or even perceive smoking and drinking as socially acceptable, could act as influential factors in encouraging adolescents to engage in these behaviors [33,34]. In many cultures, both behaviors are viewed as rites of passage that expose adolescents to other available illicit substances [35].

Psychological factors also play a role. Many adolescents resort to smoking or drinking as coping mechanisms for stress or emotional turmoil [36,37]. These initial coping behaviors may prompt adolescents to seek stronger stimuli for more intense experiences, potentially resulting in substance addiction or misuse [38].

This study further emphasized the relationship between BMI and substance use, as another predictor of adolescent substance use. From a physiological perspective, substances can modulate metabolic rates and appetite. Adolescents engaged in substance use might experience weight changes, either due to the direct effects of the substance or inconsistent dietary habits [39]. Additionally, the role of BMI extends beyond mere physiological changes. Adolescents with “nonstandard” BMIs often face societal challenges such as weight-centric bullying and entrenched societal ideals regarding body standards. The prevailing societal standards of an ideal body may influence some adolescents toward substance use, either as a means to conform to societal expectations or to address the associated mental distress [40].

Another noteworthy observation was the understated importance of academic achievement and stress status. Although stress is traditionally considered influential in adolescent behaviors [41,42], its limited representation in this study may be attributed to data constraints. These variables were absent in our extravalidation dataset, and we resorted to imputing these values using the median from our primary training cohort. This modification might have contributed to its reduced importance in our results.

### Clinical and Policy Implications

The results of this study offer significant insights into both clinical and political implications. We underscore the vital role of factors such as smoking status, BMI, and alcoholic consumption in predicting substance use among adolescents. These critical determinants enable clinicians to identify and monitor at-risk adolescents more effectively, assisting in their decision-making process [43]. Following further refinement, this model has potential commercial viability [44], especially when combined with a streamlined self-report questionnaire. The existence of multiple models assessing substance use further attests to the commercial potential of our model [45]. Emphasizing characteristics predictive of substance use is essential, suggesting the need for systems to alert parents about potential risks their children might face. Since parental intervention has proven to be effective in preventing adolescent substance use [46], establishing an early detection system becomes paramount.

### Strengths and Limitations

Findings from this study must be interpreted in light of several limitations. The external validation datasets contained numerous missing values, which could have impacted the predictive accuracy of the model. Specifically, we were unable to find corresponding data for variables like BMI (due to the absence of height and weight) and academic achievement in external cohorts such as Ungdata. Moreover, stress status, suicidal thinking, and suicide attempts were also missing or unmatched in datasets like YRBS and Ungdata, leading to gaps in these key areas. To address this gap, we applied imputation methods, including median imputation based on the KYRBS dataset for entirely missing variables [47]. While this approach mitigated the issue of missing data, it may have introduced biases, highlighting the need for more harmonized and comprehensive data collection protocols in future studies to enhance model generalizability and robustness. Additionally, this study used a discovery dataset derived from adolescents in South Korea. This biased discovery dataset could unexpectedly reflect the specific racial and cultural features unique to Korean adolescents. While our model underwent external validation from a diverse cultural and demographical landscape, we also acknowledge that it may reduce sample diversity and potentially cause overfitting issues [48]. Furthermore, this study did not pinpoint a definitive causal link between the significant risk factors and adolescent substance use. In other words, it remains unclear whether substance use influences other factors or if those factors stimulate substance use. Thus, further comprehensive studies are needed to elucidate this intricate cause-and-effect relationship. Another limitation of our study is the potential variability in feature importance rankings across different ML algorithms. Different algorithms may prioritize predictors differently due to their inherent characteristics and methods of handling data. This variability suggests that the identified predictors, such as smoking status, BMI, and alcohol consumption, should not be generalized as the sole determinants of adolescent substance use. Instead, these results should be interpreted with caution, and further studies are needed to validate the findings across diverse models and datasets. Finally, this study identifies factors associated with current and past substance use rather than explicitly predicting future trends. While the model provides valuable insights into risk factors and enables early intervention strategies, its predictive performance may be limited by the imbalanced nature of the dataset and the lack of longitudinal data. Future studies should address these limitations by incorporating balanced datasets and temporal data to enhance predictive accuracy and generalizability.

Despite these limitations, this study offers significant contributions. By using extensive datasets from South Korea, the United States, and Norway, our ML model boasts enhanced prediction accuracy, highlighting its global relevance and robustness [49]. Our strategic phased validation approach, beginning with the YRBS and progressing to the distinct Ungdata from Norway, underlines the model’s versatility across diverse sociocultural backgrounds. This phased validation not only ensures consistent model evaluation but also establishes its capability in different cultural contexts [45,50,51]. Moreover, the features incorporated into the model are derived from simple questionnaires. The primary advantage of our model-based platform is its exceptional accessibility, allowing users to gather insights through straightforward surveys. This ease enables swift evaluations and widens its scope of use, equipping both clinicians and individuals with valuable insights conveniently. Our findings provide the relative importance of numerous factors. These results can guide the decision-making process by identifying key areas for the prevention of substance use among adolescents.

### Conclusions

This study introduced an ML model using data from three distinct national cohorts to predict adolescent substance use. Among six unique predictive models, the XGBoost model consistently revealed a notable performance (AUROC: KYRBS, 80.61% [discovery]; YRBS, 79.30% [extravalidation]; and Ungdata, 76.39% [extravalidation], and precision: KYRBS, 30.42% [discovery]; YRBS, 68.37% [extravalidation]; and Ungdata, 12.74% [extravalidation]). Feature importance analysis identified smoking status, BMI, and suicidal thinking as significant contributors to the risk of substance use. Further insights into the influence of these variables were derived from SHAP value analysis, which identified smoking status, alcoholic consumption, and sadness and despair as the most impactful factors, in that order. The findings of this study indicate the potential of ML-driven predictive models to swiftly predict the likelihood of substance use among adolescents using a simplistic survey. It is anticipated that with further refinement and development, these models could be broadly used as efficient tools for preventing adolescent substance use.

## Appendix

Multimedia Appendix 1 Additional material.

## Abbreviations

AUPRC: area under precision-recall curve
AUROC: area under receiver operating characteristic curve
CDC: Centers for Disease Control and Prevention
KDCA: Korean Disease Control and Prevention Agency
KYRBS: Korea Youth Risk Behavior Web-Based Survey
ML: machine learning
NOVA: Norwegian Social Research Institute
SHAP: SHapley Additive exPlanation
TRIPOD: Transparent Reporting of a multivariable prediction model for Individual Prognosis or Diagnosis
YRBS: Youth Risk Behavior Survey

## Data Availability

The datasets generated during or analyzed during this study are available from the corresponding author upon reasonable request. The study protocol and statistical code are available from DKY. The dataset can be accessed from the Korean Disease Control and Prevention Agency, US Centers for Disease Control and Prevention, and Norwegian Social Research Institute (NOVA) through a data use agreement.
