# Supplementary material for: Machine Learning–Based Prediction of Substance Use in Adolescents in Three Independent Worldwide Cohorts: Algorithm Development and Validation Study
Source: J Med Internet Res. 2025 Feb 24;27:e62805. doi: 10.2196/62805 (PMC11894353; doi:10.2196/62805)
Supplement: Multimedia Appendix 1 [file jmir_v27i1e62805_app1.docx]

| **Supplementary Material** |
| --- |

Original Article

# Machine learning-based prediction of substance use in adolescents in 3 independent worldwide cohorts: Algorithm development and validation study

Running title: **Machine learning and substance use**

Soeun Kim,^1,2∥^ Hyejun Kim,^1,3∥^ Seokjun Kim,^1,4∥^ Hojae Lee,^1^ Ahmed Hammoodi,^5^ Yujin Choi,^1,6^ Hyeon Jin Kim,^1,2^ Lee Smith,^7^ Min Seo Kim,^8^ Guillaume Fond,^9^ Laurent Boyer,^9^ Sung Wook Baik,^10^ Hayeon Lee^1,11^, Jaeyu Park,^1,2^ Rosie Kwon,^1*^ Selin Woo,^1,4*^ Dong Keon Yon^1,2,4,12*^

^∥^ These authors contributed equally.

***Corresponding authors**

Rosie Kwon, PhD

Center for Digital Health, Medical Science Research Institute, Kyung Hee University College of Medicine, 23 Kyungheedae-ro, Dongdaemun-gu, Seoul 02447, South Korea.

Email: rosiekwon514@gmail.com

Selin Woo, PhD

Center for Digital Health, Medical Science Research Institute, Kyung Hee University College of Medicine, 23 Kyungheedae-ro, Dongdaemun-gu, Seoul 02447, South Korea.

Email: dntpfls@naver.com

Dong Keon Yon, MD, PhD, FACAAI, FAAAAI

Center for Digital Health, Medical Science Research Institute, Kyung Hee University College of Medicine, 23 Kyungheedae-ro, Dongdaemun-gu, Seoul 02447, South Korea.

Tel: +82-2-6935-2476

Fax: +82-504-478-0201

Email: [yonkkang@gmail.com](mailto:yonkkang@gmail.com)

**Contents of Supplementary appendix**

| Figure S1 | Compare the baseline feature distributions across KYRBS, YRBS, and Ungdata. | P3 |
| --- | --- | --- |
| Figure S2 | Deployed web-based application to provide substance usage prediction among adolescents: | P4 |
| Table S1 | Hyperparameters selected for the final XGBoost model | P5 |

**Figure S1.** Compare the baseline feature distributions across KYRBS, YRBS, and Ungdata. Abbreviations: BMI, body mass index.

**
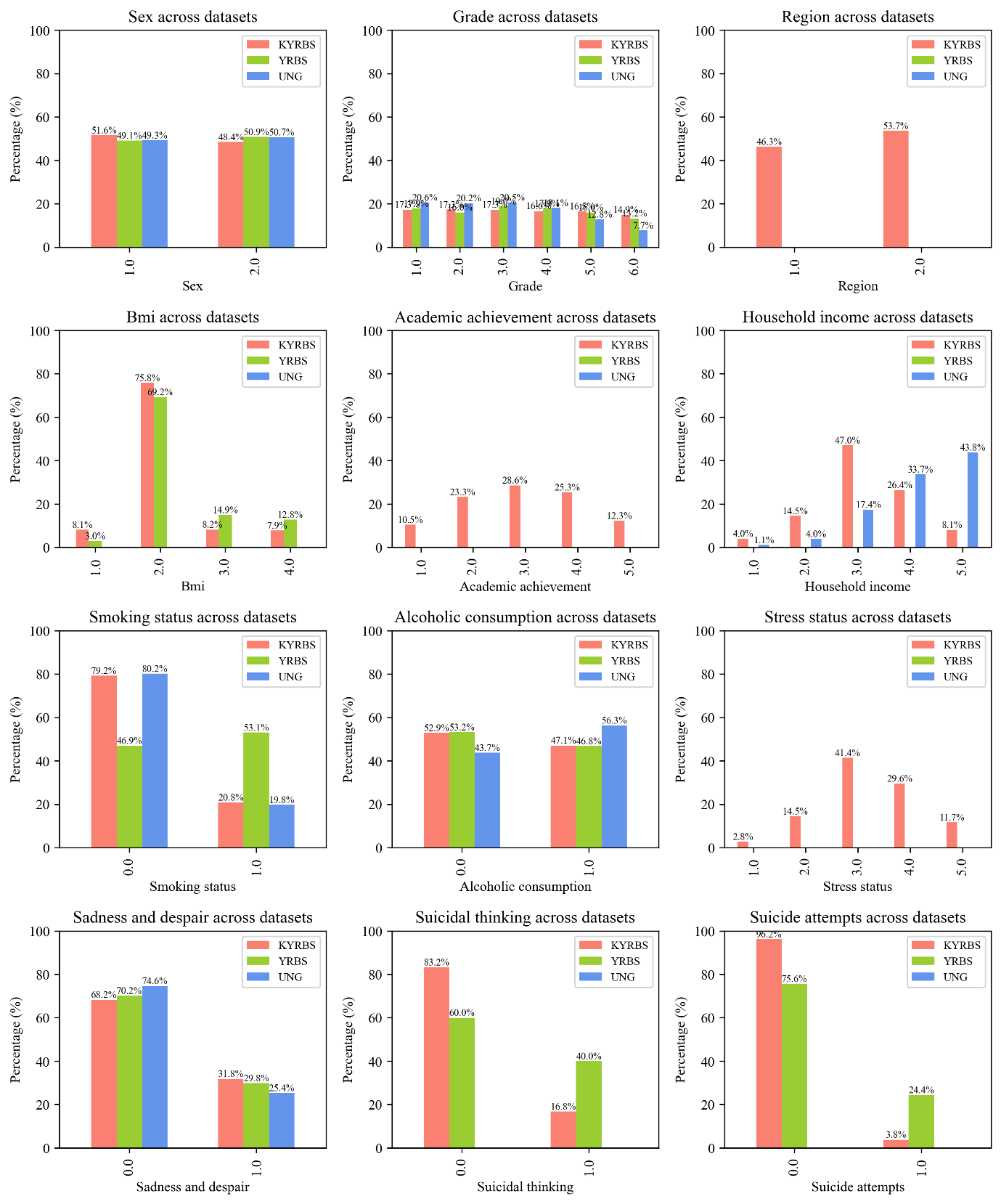
**

**Figure S2.** Deployed web-based application to provide substance usage prediction among adolescents: a web interface of user to enter information and the prediction results with the probability of substance use among adolescents. Abbreviations: BMI, body mass index.


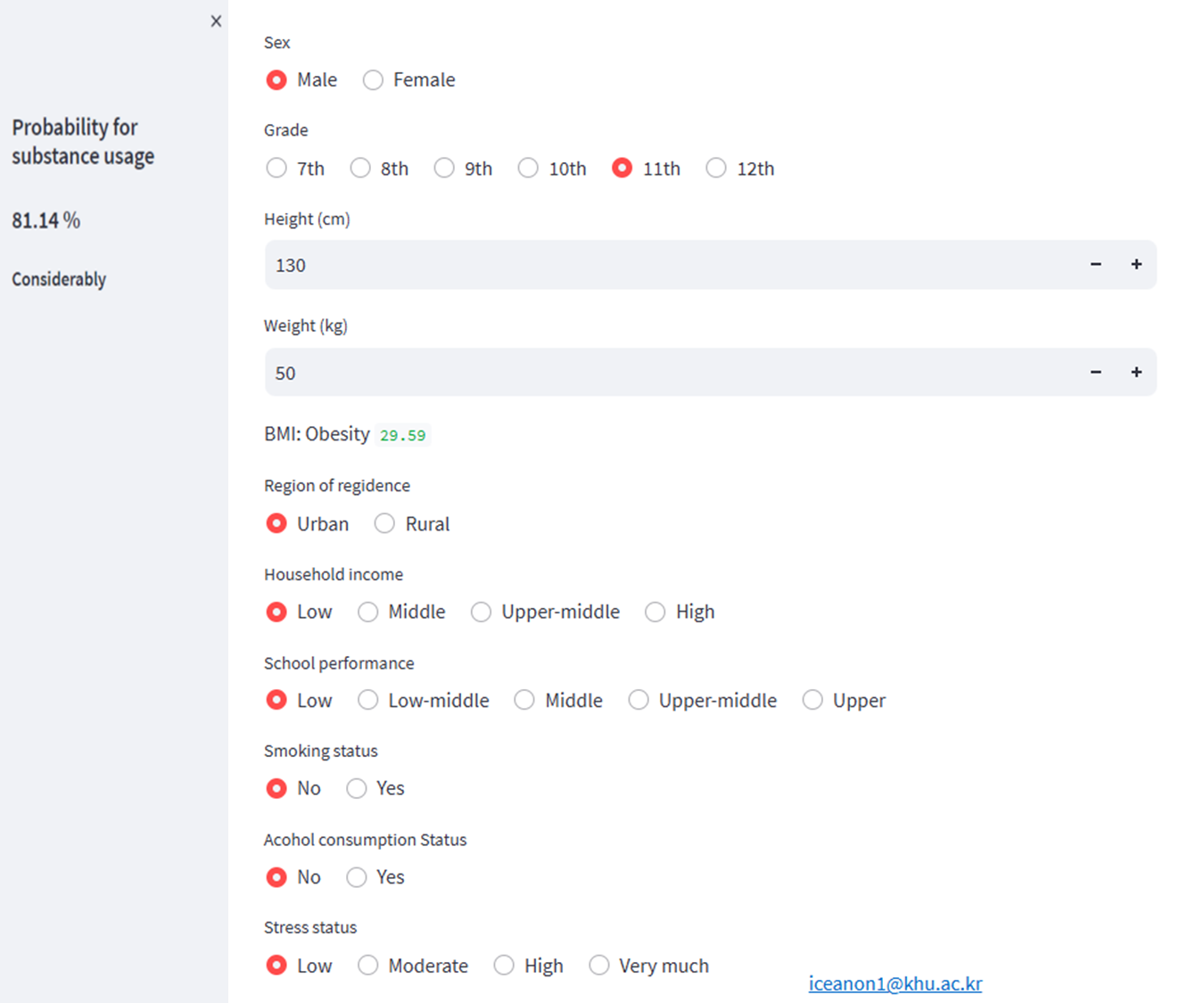


**Table S1**. Hyperparameters selected for the final XGBoost model

| Hyperparameters | Value |
| --- | --- |
| Learning rate | 0.15 |
| Max depth | 4 |
| N estimators | 100 |
| Scale pos weight | 1 |
| Subsample | 0.8 |
